# Trends in and Factors Contributing to the Slowdown in Medicare Spending Growth, 2007-2018

**DOI:** 10.1001/jamahealthforum.2022.4475

**Published:** 2022-12-02

**Authors:** Melinda B. Buntin, Salama S. Freed, Pikki Lai, Klara Lou, Laura M. Keohane

**Affiliations:** 1 Department of Health Policy, Vanderbilt University School of Medicine, Nashville, Tennessee; 2 Department of Health Policy and Management, Milken Institute School of Public Health, The George Washington University, Washington, DC; 3 Vanderbilt University School of Medicine, Nashville, Tennessee

## Abstract

**Importance:**

After decades of rapid increase, Medicare per-beneficiary spending growth was historically low in the period leading up to the passage of the Affordable Care Act. In the years immediately following the legislation, Medicare expenditure growth slowed even further.

**Objective:**

To evaluate factors contributing to the slowdown in Medicare per-beneficiary spending growth.

**Design, Setting, and Participants:**

In this cross-sectional study, expected spending growth for 2012 to 2015 and 2016 to 2018 was predicted holding payment rates and population characteristics constant. By contrasting predicted and actual spending growth during these periods, the contribution of population vs payment factors to the Medicare spending slowdown was determined. Analyses included all Medicare fee-for-service beneficiaries aged 65 years and older, ranging from 30 to 35 million beneficiaries annually between 2007 and 2018. Data analyses were conducted from January 2018 to August 2018 and updated with new data in June 2021.

**Main Outcomes and Measures:**

The main outcome included annual growth in total per-beneficiary spending. The roles of payment rate changes and differences in the Medicare population over time were considered, including demographic characteristics and numbers of chronic conditions.

**Results:**

Between 2008 to 2011 and 2012 to 2015, the adjusted annual Medicare Parts A and B per-beneficiary spending growth rate declined from 3.3% to −0.1%. From 2016 to 2018, the mean annual Medicare spending growth rate rose relative to the previous period but remained lower than in the baseline period at 1.7% per year. This slowdown extended across all sectors within Parts A and B, except for physician-administered drugs offered under Part B. Changes in payment rates (including sequestration measures) and beneficiary characteristics explained 44% of the difference in overall per-beneficiary spending growth between 2007 to 2011 and 2012 to 2015, and 63% between 2007 to 2011 and 2016 to 2018.

**Conclusions and Relevance:**

In this cross-sectional study of trends in spending growth per Medicare beneficiary aged 65 years or older, results suggested that Medicare payment policy, including sector-specific payment rate changes and sequestration, will be a critical determinant of whether the Medicare spending growth slowdown persists.

## Key Points

**Question:**

Which factors contributed to the decline in Medicare per-beneficiary spending growth over the past decade?

**Findings:**

In this analysis of individual-level Medicare spending data of more than 30 million beneficiaries, 44% of the decline in per-beneficiary spending growth from 2012 to 2015 and 63% from 2016 to 2018 could be attributed to lower increases in payment rates, sequestration measures, and shifts in beneficiary characteristics.

**Meaning:**

Continued attention to Medicare payment policies and how to target them appropriately will be needed to maintain slow spending growth and extend the Medicare program’s sustainability.

## Teaser

This cross-sectional study evaluates factors contributing to the slowdown in Medicare per-beneficiary spending growth.

## Introduction

For most of the program’s history, Medicare spending has outpaced economic growth, creating challenges for sustaining the program financially without increasing tax revenues, cutting benefits, or reducing payments for care delivered. However, Medicare per-beneficiary spending growth slowed throughout the mid-2000s to late-2000s; it reached historically low rates of growth at the beginning of the past decade (2010 to 2015).^1^ Medicare Parts A and B annual per-beneficiary spending growth remains low, and peaked in 2018 at 3.0% before slowing again in 2019 to 2.4%.^2,3^

Although health spending growth more than doubled in the first year of the COVID-19 pandemic,^4^ slower growth continued through the pandemic and growth currently remains modestly above its prepandemic rate and steady in recent months.^5^ Due to the COVID-19 pandemic, demand for Medicare services in 2020 was low, but Medicare payments were accelerated.^1,6^ This period of slower Medicare spending growth invites debate about whether these trends will persist long after the pandemic, a question that is difficult to answer without understanding the causes of the spending slowdown leading up to 2020^7,8,9,10^ and the economic effects of the COVID-19 pandemic on Medicare.^11,12^ Knowing which factors account for the Medicare growth slowdown—and how policy makers might be able to sustain them—is crucially important, especially now as the Hospital Trust Fund runs the risk of insolvency by 2028.^1^

Broadly speaking, per-beneficiary spending growth is driven by changes in payment rates and the quantity of services reimbursed by Medicare. The first factor, payment policy, has been one of the major policy levers to control Medicare costs. Most notably, introducing prospective payments for inpatient care and postacute services resulted in spending declines in these sectors.^12,13,14,15^ More recently, the Affordable Care Act reduced the growth of Medicare payment rates for some sectors and launched multiple payment demonstrations aimed at decreasing unnecessary medical spending.^16,17^

The second component of spending growth, changes in the volume of services reimbursed by Medicare, can reflect multiple factors. One well-established driver of spending growth, technological innovation, has historically expanded the range of treatable conditions and increased the overall number of services delivered to Medicare beneficiaries.^7,18^ Reimbursement levels can also incentivize changes in diagnosis patterns and frequency. Recent evidence suggests that rising volumes of highly reimbursed diagnosis-related groups are driving increases in Medicare spending for inpatient services.^19^ In the absence of new technology, the Medicare population’s demand for health care services can also change over time. As the Baby Boom generation continues to age into Medicare, the characteristics of the average Medicare beneficiary are, for now, shifting to a younger demographic profile.^20^ Moreover, compared with previous generations, the prepandemic US population experienced delayed onset of disabling conditions and longer lifespans.^21,22,23,24^ Such patterns influence Medicare beneficiaries’ health and the volume of health care services subsequently provided.

This study considers changes in Medicare payment rates, and other factors that may influence utilization, to understand and quantify the reasons behind slowed Medicare spending among beneficiaries aged 65 years and older, a topic of interest given the threat of the Medicare Hospital Trust Fund’s insolvency. The focus of this study is on Medicare program spending between 2007 and 2018. This study describes how Medicare spending growth changed across service sectors and examines how Medicare Parts A and B per-beneficiary spending growth may have been influenced by changes in sector-specific payment rates and beneficiary traits over time.

## Methods

The Vanderbilt University Medical Center institutional review board exempted this cross-sectional study from review and waived informed consent because all data were deidentified. This study followed the Strengthening the Reporting of Observational Studies in Epidemiology (STROBE) reporting guideline.^25^ Additional information on this study’s STROBE compliance can be found in eMethods in the Supplement. Data analyses were conducted in SAS statistical software, Enterprise Version V7.1 (SAS Institute Inc) from January 2018 to August 2018 and updated with new data in June 2021.

### Study Data and Population

In this cross-sectional study, the Medicare Master Beneficiary Summary File for 2007 to 2018 was used to provide individual-level data on annual health care costs and utilization by sector for Medicare beneficiaries, which ranged from approximately 30 to 35 million beneficiaries annually. We also analyzed Medicare Part A claims to identify inpatient rehabilitation facility services and categorize them as postacute services instead of inpatient treatment. The Medicare Master Beneficiary Summary File data identified whether beneficiaries had any of 26 common chronic conditions, as measured by claims-based algorithms (full list of conditions included in eTable 1 in the Supplement).^26^

Analyses included all Medicare beneficiaries aged 65 years and older enrolled in Parts A or B and not enrolled in Medicare Advantage in July of each year. We looked at those with A or B coverage to capture costs for all beneficiaries not covered by Medicare Advantage. New Medicare beneficiaries and decedents who did not have Medicare benefits in July were excluded if they had Medicare Advantage in their first or last month of Medicare benefits, respectively.

### Descriptive Analyses

The primary outcome, growth in Medicare per-beneficiary spending, included all Parts A and B payments made by the Medicare program, net of any cost-sharing. The average annual percentage change was examined in Medicare per-beneficiary spending overall and by service sector for the period 2007 to 2018, with the latter results broken down into 3 periods to better illustrate the patterns over time. Note that in the following sections annual growth rates will be associated with the second year in each interval (ie, growth from 2007 to 2008 will be labeled 2008).

### Spending Growth Decomposition Analyses

The analysis examined several potential contributors to changes in average Parts A and B per-beneficiary spending growth. As described in eMethods in the Supplement, to quantify how much of the decline in spending growth can be attributed to observed factors, the rate of Medicare spending growth was first contrasted before and after adjusting annual spending levels for factors related to Medicare payment policy. The rates of growth in payments are contrasted with the Consumer Price Index for All Urban Consumers (CPI-U), a measure of general inflation.

Changes were also examined accounting for characteristics that could influence beneficiaries’ use of services: age (categorized as ages 65-69, 70-74, 75-79, 80-84, and ≥85 years), sex, number of chronic conditions (none, 1-3, ≥4), and having Part A only coverage. Because there could be correlation between the factors that influence beneficiaries’ use of services, adjustments for these factors were applied sequentially in the order listed previously. For example, spending growth estimates associated with changes in the proportion of female Medicare beneficiaries reflect previous adjustments for Medicare payment changes and changes in the age distribution of the Medicare population. These analyses show what Medicare spending growth would have been if beneficiary characteristics and Medicare payment rate levels were held constant over time.

## Results

**Figure 1.**
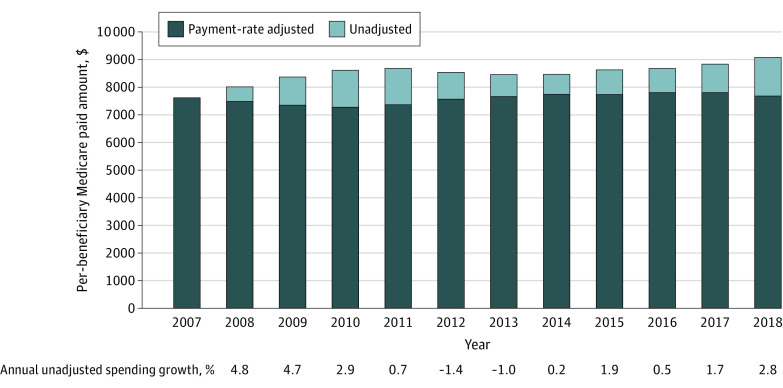
Medicare Per-Beneficiary Spending Totals and Spending Growth, 2007-2018 This analysis included all Medicare Parts A and B payments for fee-for-service beneficiaries aged 65 years and older who were not enrolled in a Medicare Advantage plan in July of each sample year. Adjusted figures represent mean spending per beneficiary after adjusting for payment rate increases since 2007 and sequestration measures that took effect in 2013 and remained active through the end of the study period in 2018. Source: Authors’ calculations using 100% data from the Medicare Master Beneficiary Summary File.

Unadjusted Medicare Parts A and B per-beneficiary spending for Medicare beneficiaries aged 65 years and older grew at an average rate of 3.3% per year for the period of 2008 to 2011 (Figure 1, Table). For the period of 2012 to 2015, per-beneficiary spending growth declined −0.1% per year, before increasing again at an average rate of 1.7% per year for the period of 2016 to 2018. In terms of spending levels, mean Medicare Parts A and B spending per-beneficiary increased from $7669 in 2007 to $9129 in 2018. If payment rates had remained constant at 2007 levels, overall Parts A and B per-beneficiary spending would have been $7744 in 2018, or 17.9% lower than actual 2018 spending (Figure 1). Characteristics of the more than 30 million traditional Medicare beneficiaries per year included in this study can be found in eTable 2 in the Supplement. The beneficiary population is approximately 45% male and 55% female, with the highest percentage share (32%) in the age 65 to 69 years age group.

**Table.** Contributions to Annual Growth in Per-Beneficiary Spending in Parts A and B of Medicare^a^

Variable	Period 1, 2008-2011	Period 2, 2012-2015	Period 3, 2016-2018	Differences	
				2012-2015	2016-2018
Annual spending growth (per FFS beneficiary)	3.3	−0.1	1.7	−3.4	−1.6
Potential contributors to the slowdown					
Growth in payment rates	2.4	1.6	1.5	−0.8	−1.0
Payment reductions under sequestration	0.0	−0.5	0.0	−0.5	0.0
Growth in demand by beneficiaries					
Changes in age	−0.4	−0.7	−0.3	−0.3	0.1
Changes in % female	0.0	0.0	0.0	0.0	0.0
Changes in chronic conditions^b^	−0.4	−0.3	−0.5	0.1	−0.1
Changes in % with only part A	0.0	0.0	0.0	0.0	0.0
Difference in spending growth explained by factors above	NA	NA	NA	−1.5	−1.0

Abbreviations: FFS, fee-for-service; NA, not applicable.

^a^ Source: Authors’ calculations using *Federal Register* filings (for payment rate changes), information on sequestration measures in place since April 2013, and the 100% Master Beneficiary Summary File (for demand variables). The analysis covers spending for fee-for-service beneficiaries aged 65 and older from 2007 to 2018. Differences may not sum perfectly due to rounding.

^b^ Changes in chronic conditions reflect changes in the average number of per-beneficiary chronic conditions, grouped into 3 categories: 0, 1-3, and ≥4.

**Figure 2.**
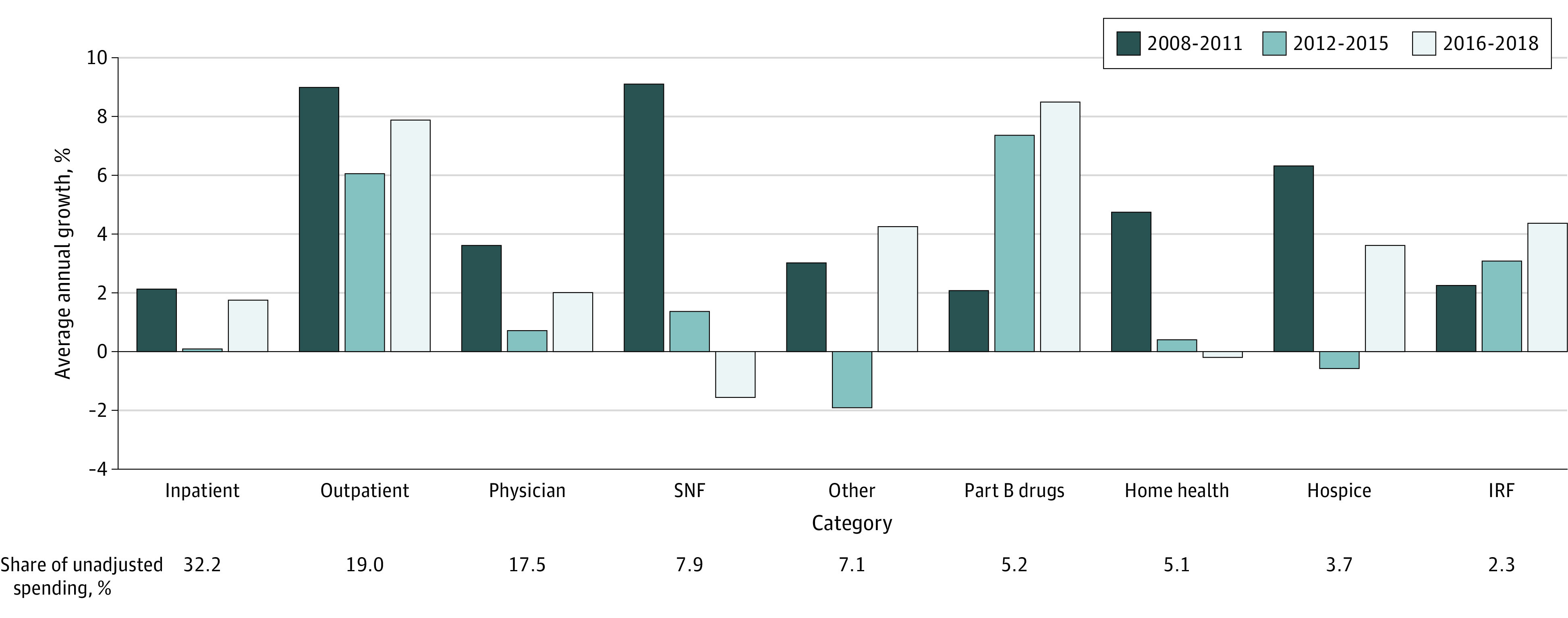
Average Annual Growth in Medicare Parts A and B Per-Beneficiary Spending by Service Sector, 2008-2011 Compared With 2012-2015 and 2016-2018 Bars are sorted based on each service’s share (%) of spending in 2018, from largest (inpatient, 27.7%) to smallest (IRF, 2.0%). The physician category includes all services performed in a physician’s office. The other category combines ambulatory surgical centers, dialysis centers, tests, durable medical equipment, and nonphysician carrier claim payments, such as ambulances and chiropractor services. IRF indicates inpatient rehabilitation facilities; SNF, skilled nursing facilities. Source: Authors’ calculations using 100% Master Beneficiary Summary File information for fee-for-service beneficiaries aged 65 and older.

Figure 2 shows average Medicare Parts A and B per-beneficiary spending for each service sector. Almost every Medicare sector experienced a slowdown in spending growth in the years 2012 to 2015 and 2016 to 2018 compared with growth in the years 2008 to 2011, though the slowdown was less pronounced in the 2016 to 2018 period. A majority of the health care service sectors had lower spending growth in the second and third periods than in the first. In contrast to spending growth trends in other sectors, spending for Part B drugs and inpatient rehabilitation facilities grew at a higher rate in the years 2012 to 2015 and 2016 to 2018 than in the 2008 to 2011 period.

### Contribution of Medicare Payment Rate Changes

**Figure 3.**
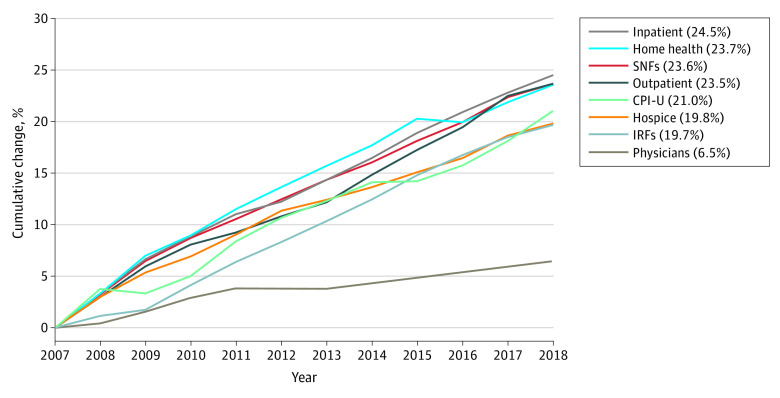
Medicare Payment Rate Increases—Total and Select Service Sectors, 2007-2018 Each sector’s specific price index was applied to the total spending in that sector. Total cumulative payment rate updates were then calculated by summing all the payment-rate adjusted sector totals. The Consumer Price Index for All Urban Consumers (CPI-U) was used to update payment rates. Skilled nursing facilities (SNFs), home health agencies, and inpatient rehabilitation facilities (IRFs) constitute postacute care. Source: Payment rate updates are abstracted from *Federal Register* announcements and include Affordable Care Act–mandated reductions, productivity adjustments, and any other reductions mandated by Congress or the Centers for Medicare & Medicaid Services (see eMethods in the Supplement).

Inpatient (24.5%) and physician (6.5%) services had the largest and smallest cumulative increases in payment rates, respectively, from 2007 to 2018 (Figure 3). Payment increases for postacute care sectors were similar: home health agencies (23.7%), skilled nursing facilities (23.6%), and inpatient rehabilitation facility (19.7%). The CPI-U, a measure of general inflation, grew 21.0% over this period—considerably faster than payments for physicians’ services but on par with payment growth for institutional services. The Part B drug price index increased more than 90% during this study period; because this index is not directly comparable with the other sectors, it is not included in Figure 3 (see eMethods in the Supplement for details).

**Figure 4.**
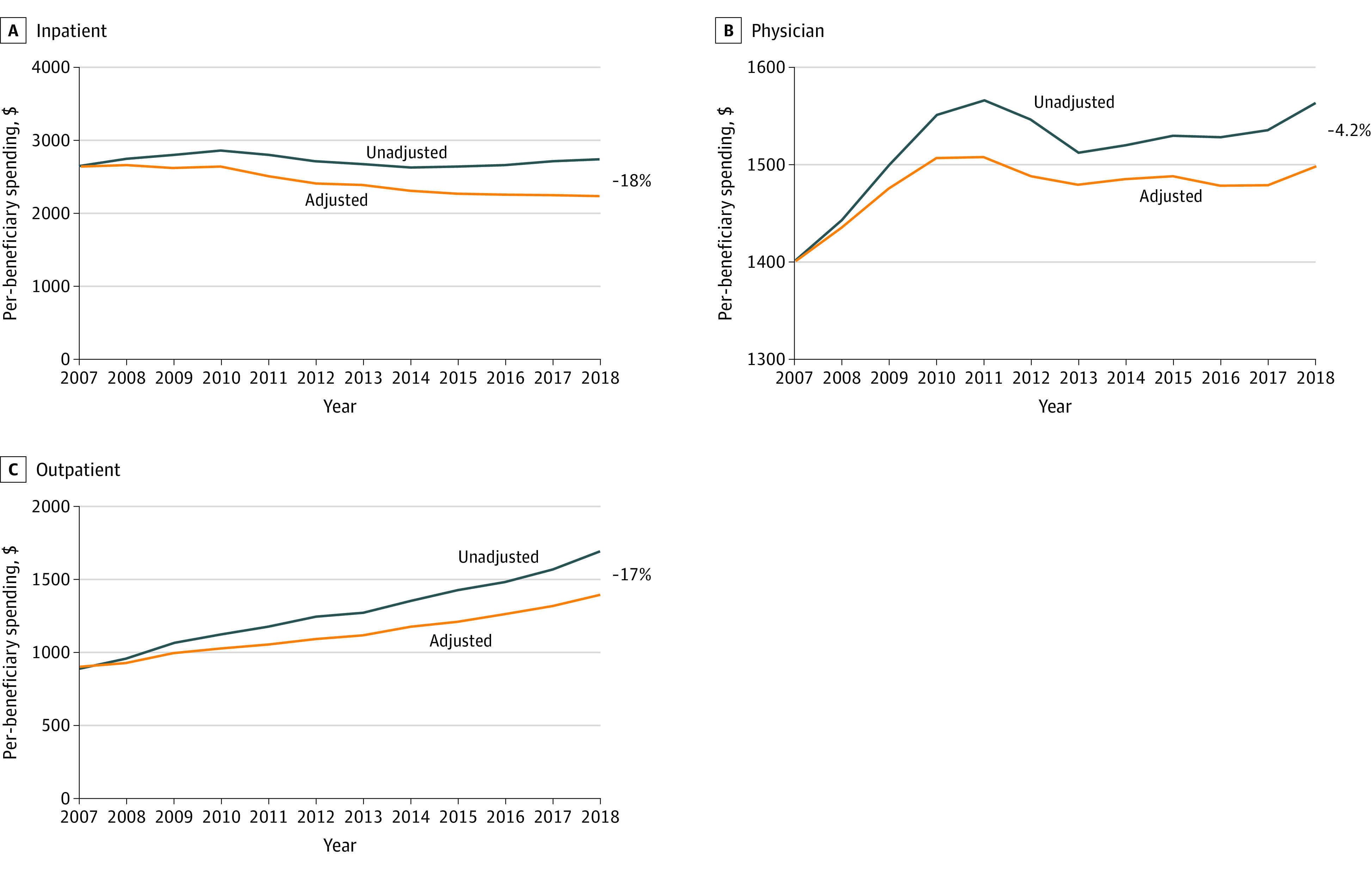
Medicare Per-Beneficiary Spending by Service Sector, 2007-2018 Source: Authors’ calculations using 100% Master Beneficiary Summary File information for all fee-for-service beneficiaries aged 65 years and older and payment rate updates abstracted from *Federal Register* announcements (see eMethods in the Supplement).

Figure 4 displays per-beneficiary spending levels for the 3 largest service sectors—comprising 69% of Medicare Parts A and B spending in 2018—with and without adjusting for payment rate changes. If payment rates had not changed since 2007, inpatient per-beneficiary spending would have been $2254 in 2018, or 18.0% lower than actual 2018 spending. Instead, inpatient per-beneficiary spending in 2018 was $2749, or 3.6% higher than the 2007 spending levels. Per-beneficiary spending on outpatient services increased 90.4% between 2007 and 2018, from $889 to $1692. Without payment changes over this time, per-beneficiary outpatient spending would have been $1397 in 2018, or 17.4% lower than actual spending. Per-beneficiary spending for physician services increased by 11.5% from $1401 in 2007 to $1562 in 2018. Had payment rates not increased, per-beneficiary spending on physician services would have been $1497 or 4.2% lower than 2018 actual spending.

### Factors Underlying Medicare Per-Beneficiary Spending Growth

Of the 3.4 percentage point (3.3% vs −0.1%) difference in per-beneficiary spending growth in Medicare Parts A and B between 2008 to 2011 and 2012 to 2015 (Table), changes in payment rates and beneficiary characteristics that could influence use of medical services accounted for 1.5 percentage points of this decline. In relative terms, these factors explained 44% (−1.5/−3.4 = 0.44) of the observed slowdown (Table). The difference in per-beneficiary spending growth between 2008 to 2011 and 2016 to 2018 was 1.6 percentage points. Payment rates and beneficiary characteristics accounted for a larger fraction of this change (−1.0/−1.6 = 0.63 or 63%). A detailed example of this calculation is provided in eMethods in the Supplement.

If payment rates had stayed constant at 2007 levels, spending growth would have still been lower than observed, especially in the years 2008 to 2011 (Table). The spending growth rate was 2.4 percentage points, 1.6 percentage points, and 1.5 percentage points higher in the first, second and third periods, respectively, due to payment rate increases. In other words, payment rate changes increased spending growth more in the first period than during the second and third periods. This difference in the payment rate changes accounted for 0.8 percentage points, or 24% of the slowdown in spending growth rates between 2008 to 2011 and 2012 to 2015, and 1.0 percentage point, or 63%, of the slowdown between the first and third periods.

Sequestration measures took effect in 2013 and remained active at the end of this study period in 2018.^27^ These measures reduced Medicare payment levels by 2% (but did not alter premiums for Medicare Parts B and D, cost sharing, Part D subsidies, and Part A trust-fund revenues).^28^ In terms of spending growth, sequestration represents one abrupt change in payment levels, not annual adjustments to payment growth rates that compounded over time: sequestration measures reduced mean spending growth for 2012 to 2015 by 0.5 percentage points, or 15% of the slowdown in spending growth rates between 2008 to 2011 and 2012 to 2015.

Factors related to beneficiaries’ use of health services were modestly associated with spending growth slowdown than payment-related factors. Over the study period, the share of Medicare beneficiaries under age 70 years increased with the aging of the Baby Boom population into Medicare, as did the share of beneficiaries with no chronic conditions. Without shifts in spending associated with age, Medicare spending growth rates would have been higher in all 3 time periods, but particularly in the 2012 to 2015 period. Without changes in spending associated with chronic conditions, Medicare spending growth rates also would have been higher in all 3 time periods. Accounting for both these changes in the population narrows the difference in spending growth rates by 0.2 percentage points, or 6% of the slowdown in spending growth rates between 2008 to 2011 and 2012 to 2015.

## Discussion

This cross-sectional study expanded upon previous research that focused on the initial downturn in Medicare spending growth in the late 2000s.^29^ Because of the coincident timing, the Great Recession was considered a leading cause of this health spending slowdown.^7,8,9,10,30^ Although there is evidence supporting this theory in commercial insurance,^31,32^ there is little evidence that Medicare beneficiaries cut back on medical spending in response to the economic downturn.^31,33^ In fact, Medicare spending growth remained low even as the economy recovered. Between 2007 and 2018, Parts A and B per-beneficiary spending increased $1460, from $7669 to $9129. We found overall growth slowed from 3.3% during the 2008 to 2011 period to −0.1% during the 2012 to 2015 period. During the 2016 to 2018 period, growth was higher than that observed in the 2012 to 2015 period, but growth was still lower than that in the 2008 to 2011 period. This pattern was pervasive across the inpatient, physician, and outpatient sectors and consistent with CBO’s reports that overall *additional cost growth*—or per capita spending in excess of per capita GDP growth—in Medicare averaged approximately −0.1% from 2005 to 2017.^34^

The present study’s decomposition analysis accounted for 44% of the decline in Medicare Parts A and B per-beneficiary spending growth between 2008 to 2011 and 2012 to 2015 periods and 63% of the decline between 2008 to 2011 and 2016 to 2018. Only 6% of the slowdown was due to changes in the beneficiaries’ age composition and lower spending on chronic conditions in the 2012 to 2015 period, and beneficiary characteristics accounted for none of the slowdown from 2016 to 2018. The remainder of the observed spending slowdown was attributed to changes in payment rates (24% in 2012 to 2015, 63% in 2016 to 2018) and federal government sequestration (15% in 2012 to 2015). These results reflect a stronger role of payment rate changes in the per-beneficiary spending growth slowdown, relative to the findings of previous literature about how payment rate changes influence spending, reflecting the broader scope of the payment and sequestration measures during recent years.^8,9^ They are also broadly consistent with the longstanding literature on the role of medical technology in medical spending growth, which finds that, after accounting for the directly measurable contributors to cost growth, “the enhanced capabilities of medicine most likely account for the bulk of the increase.”^18^

Payment rate changes had varying influences on overall spending growth and on growth in the largest service sectors (inpatient, outpatient, and physician services). While inpatient and outpatient services enjoyed payment rate increases of 24.5% and 23.5% between 2008 and 2018, respectively, the payment rate increase for physician services (6.5%) was much lower. Inpatient and outpatient payment changes reflect both the increasing cost of service provision and payment rate reductions enacted under the Affordable Care Act. These findings are consistent with a similar analysis of private insurance payment rate changes for hospital-based inpatient and outpatient care^33,35^ in showing that payments for these services grew substantially faster than those for physician services. In addition, the findings of this study are also consistent with other studies of the role on inpatient service use.^36^ Medicare payment rates for physician services were essentially frozen after the end of the Sustainable Growth Rate payment system by the Medicare Access and CHIP (Children’s Health Insurance Program) Reauthorization Act of 2015 (MACRA) legislation until new incentive payment systems were implemented in 2019.^37^

In addition to directly slowing Medicare spending growth, stagnant payment rates may change the incentive to deliver services. The literature provides a range of findings with regard to how clinicians and health care organizations respond to payment rate changes including by providing more services or changing coding^38^ to maintain their reimbursement levels, shifting services from low-cost to high-cost settings, merging or consolidating, and reducing frequency or intensity with which they treat and accept patients with Medicare.^37,39,40,41,42^ The overall outcome of low growth in physician payment rates in restraining per-beneficiary spending growth in Medicare over the past decade is a combination of the effects of these payment rate changes.

Payment rate adjustments and sequestration both contributed to slower spending growth in recent years. However, these measures differ substantially in how they influence beneficiaries, clinicians and health care organizations, and long-term spending trajectories. First, several of the payment rate reductions in the Affordable Care Act–targeted specific sectors, including high-cost services like inpatient care. In contrast, sequestration cut payments to all Medicare sectors, even those that may be cost-efficient. MedPAC has criticized sequestration for not accounting for whether each sector’s payment rates are high enough to protect beneficiaries’ access to care.^43^ Second, payment rate adjustments can be designed to generate gradual cumulative change in spending growth over several years. Sequestration measures went into full effect in 1 year, and consequently only reduced spending growth during that year. Finally, according to current law, sequestration measures only defer an increase in spending growth.^27^ Indeed, because of the COVID-19 pandemic, the full sequestration cuts of 2% were temporarily suspended beginning in May 2020 but were fully reinstated July 1, 2022. This means that claims with dates of service from May 1, 2020, through December 31, 2021, are not subject to the 2% reduction in Medicare payments. Much like a 2% off coupon that will expire, Medicare must pay full price again once the sequestration period ends in 2030 unless there is further legislation.

For policy makers seeking opportunities to contain long-term spending growth, these distinctions raise questions about the optimal approach for how payment policy can influence the Medicare budget. Targeted payment reforms, such as the implementation of prospective payments for inpatient and postacute services, can create incentives to deliver care more efficiently. These changes can be implemented gradually, allowing time for clinicians and health care organizations to adjust to new payment incentives. A disadvantage is that designing effective payment incentives is complicated by clinical and political considerations, as demonstrated by the difficult MACRA physician payment implementation.

### Limitations

This study was limited to Medicare beneficiaries aged 65 years and older and those who are not enrolled in Medicare Advantage plans. The age limitation was used because the patterns of enrollment of beneficiaries with disability changed during the recession,^44^ and it was not possible to accurately capture those changes with the age and chronic condition categories available in this study’s data. Medicare Advantage data on chronic conditions or on payment rates that applied within plans were not accessible during this study; however, it is important to note that Medicare Advantage capitation rates generally reflect the payment and utilization patterns in fee-for-service Medicare.

In addition, due to data limitations, Medicare Part D spending data at the individual level were not available. Unlike the other sectors where spending at the individual level is measured according to the amount reimbursed by Medicare, Part D spending data are based on point-of-sale payments covered by Part D plans and reported to the Centers for Medicare & Medicaid Services. Because the data do not account for rebates to plans, which are proprietary information, they may overestimate actual spending levels.^45^ Additionally, chronic condition coding may have changed due to incentives to upcode the intensity of services delivered.^38^ However, when Part D per-beneficiary spending is added to overall totals, the decline in spending growth is only slightly attenuated: mean annual growth including Part D was 3.4% for 2008 to 2011, 0.8% for 2012 to 2015, and 2.1% for 2016 to 2018 (data not shown).

Finally, only approximately 7% of beneficiaries are enrolled in Part A only, and many of those are people older than 64 years still working and covered by employer-sponsored insurance.^46^ Only approximately 7% of beneficiaries are enrolled in Part A only, and many of those are people older than 64 years still working and covered by employer-sponsored insurance.^46^ In earlier work, we found that the inclusion of Part A–only beneficiaries did not appreciably change spending growth trajectories, and we did not find any change in growth over this time period due to the changing proportion of Part A–only beneficiaries in the decomposition analysis, but this subject warrants further attention.^47^

In addition, the mechanisms driving the Medicare spending growth slowdown extend beyond the direct contributions discussed here, there are indirect contributors to slower spending growth, particularly interactions between prices and demand where clinicians and health care organizations may have expanded service volume in response to payment rates changes, which are difficult to quantify. Lower spending on chronic conditions such as cardiovascular events may also be explained by the availability and increased use of medications that control risk factors.^15,48,49^

## Conclusions

In this cross-sectional study of trends in spending growth per Medicare beneficiary aged 65 years and older, we found that payment policies have contributed substantially to slowing spending growth over the most recent decade for which data are available. These payment policies have included sequestration and payment rate updates that were lower than those in prior years. While these policies have helped to contain costs for taxpayers and those paying Medicare premiums, further research is needed to determine whether they have had adverse effects on access to or quality of care. Nonetheless, these findings suggest that the persistent threat of payment cuts, especially those that target inefficient and costly services, could be a primary force in driving long-term sustainable Medicare spending growth.

## Notes

Supplement. **eTable 1.** CCW Chronic Conditions **eMethods.** STROBE Compliance, Spending Growth Analyses, and Sample Calculation **eTable 2.** Beneficiary Demographics Characteristics (Used in Model of Demand for Services), 2007-2018
