# Supplementary material for: Trends in and Factors Contributing to the Slowdown in Medicare Spending Growth, 2007-2018
Source: JAMA Health Forum. 2022 Dec 2;3(12):e224475. doi: 10.1001/jamahealthforum.2022.4475 (PMC9719052; doi:10.1001/jamahealthforum.2022.4475)
Supplement: Supplement. — eTable 1. CCW Chronic Conditions eMethods. STROBE Compliance, Spending Growth Analyses, and Sample Calculation eTable 2. Beneficiary Demographics Characteristics (Used in Model of Demand for Services), 2007-2018 [file jamahealthforum-e224475-s001.pdf]

Supplemental Online Content

Buntin MB, Freed SS, Lai P, Lou K, Keohane LM. Trends in and factors contributing to the slowdown in Medicare spending growth, 2007-2018. *JAMA Health Forum.* 2022;3(12):e224475. doi:10.1001/jamahealthforum.2022.4475

eTable 1. CCW Chronic Conditions

eMethods. STROBE Compliance, Spending Growth Analyses, and Sample Calculation

eTable 2. Beneficiary Demographics Characteristics (Used in Model of Demand for Services), 2007-2018

This supplemental material has been provided by the authors to give readers additional information about their work.

© 2022 Buntin MB et al. *JAMA Health Forum.*

eTable 1. CCW Chronic Conditions

CCW Chronic Conditions[1]	
Acquired Hypothyroidism	Chronic Kidney Disease
Acute Myocardial Infarction	Chronic Obstructive Pulmonary Disease
Alzheimer's Disease	Depression
Alzheimer's Disease, Related Disorders, or Senile Dementia	Diabetes
Anemia	Glaucoma
Asthma	Heart Failure
Atrial Fibrillation	Hip / Pelvic Fracture
Benign Prostatic Hyperplasia	Hyperlipidemia
Cancer, Colorectal	Hypertension
Cancer, Endometrial	Ischemic Heart Disease
Cancer, Breast	Osteoporosis
Cancer, Lung	Rheumatoid Arthritis / Osteoarthritis
Cancer, Prostate	Stroke / Transient Ischemic Attack
Cataract	

eMethods: STROBE Compliance, Spending Growth Analyses, and Sample Calculation

This study includes all required items on the STROBE guidelines checklist for cross-sectional studies in the six main sections: Title and Abstract, Introductions, Methods, Results, Discussion, and Other Information The design, background, and specific objectives of the study are clearly stated. Additionally, data sources, study size (see eTable 2 for more information), and statistical methods used are outlined. Furthermore, key results, interpretation, and limitations are included in the Discussion section. Finally, funding information relating to the study is included.

Our analysis examined several potential contributors to changes in average Parts A and B per-beneficiary spending growth, net of any cost-sharing or supplemental payments, in 2012-2015 and in 2016-2018 relative to 2008-2011. To quantify how much of the decline in spending growth can be attributed to observed factors, we contrasted the rate of Medicare spending growth

© 2022 Buntin MB et al. *JAMA Health Forum.*

before and after adjusting annual spending levels for factors related to Medicare payment policy and beneficiaries' use of services.

We consider two different sources for how Medicare payment amounts changed during our study period. The first source includes annual payment rate changes for each Medicare sector, which the Centers for Medicare and Medicaid Services (CMS) calculates according to formulas known as "market basket" updates that reflect changes in the costs of providing services. These annual payment rate updates can be further modified by legislation. For example, the Affordable Care Act reduced annual payment rate adjustments in specific years for inpatient, outpatient, hospice, and home health services. This legislation also introduced a productivity adjustment that assumed Medicare providers' productivity would grow at the same rate as the broader economy, thus reducing payment rates to reflect increased productivity. Using *Federal Register* notices and VRDC data, we abstracted payment updates for 2007-2018 and constructed an annual payment index for each sector.

CMS does not calculate market basket updates for all sectors in our analyses, so we used the Consumer Price Index for All Urban Consumers (CPI-U) and the Producer Price Index for Pharmaceutical Preparation Manufacturing reported by the Bureau of Labor Statistics for the "Other" service sector and Part B Drugs indices, respectively.[2] Medicare Part B currently uses a "buy and bill" payment model, in which providers first purchase the drug and then bill for it after it is administered. Biologics, the most expensive drugs covered by Part B, constitute for 92 percent of Part B drug spending growth from 2006 to 2017[5] and culminated to approximately $145 billion in spending in 2018.[4] With this relatively small number of Part B biologic drugs accounting for most of the total Medicare payment for Part B drugs,[5] high spending and rapid

spending growth in this sector are largely driven by the lack of limits on launch prices of new drugs and on annual price increases for existing drugs.

To create an index for per-beneficiary spending overall: 1) each sector's total spending was first calculated, 2) the payment rate index for each sector was then applied to its respective sector total, and 3) all the payment-rate adjusted sector totals were summed together. A similar approach was followed to create an index for post-acute care using the skilled nursing facility, home health agency, and inpatient rehabilitation facility sectors. Changes in these indices reflect how much Medicare payment rates have increased since 2007.

The second source of change in Medicare payment amounts is federal budget sequestration measures. Under the Budget Control Act passed in 2011, Medicare payments (and funding to other federal programs) were to be reduced if the federal budget exceeded a target amount. Accordingly, for the period April 2013 through the end of the study period in 2018, all Medicare provider payments were reduced by 2%. Medicare payments for Part B drugs also dropped from Average Sales Price (ASP) plus 6% to ASP plus 4.3%. These reductions are applied after the annual payment rate adjustments are calculated for each sector, and do not apply to the cost-sharing amounts that Medicare beneficiaries must pay. Under current law that accounts for the COVID-19 pandemic, the period in which these reductions are in effect has been extended through 2030.[3]

To quantify the sources of spending growth, the annual expected change in per-beneficiary spending for each year was estimated as if all factors had remained constant at 2015 levels. Specifically, Medicare payments were deflated to 2015 levels based on sector-specific payment indices to estimate the contribution of payment rate changes. For the period sequestration was in effect, Medicare payments were inflated by 2% to estimate spending levels in the absence of

© 2022 Buntin MB et al. *JAMA Health Forum.*

sequestration. (2013 spending was only inflated by 1.5% since sequestration was only in effect for three quarters of the year). Finally, indirect adjustment methods were used to estimate what spending would have been in the absence of changes in beneficiary characteristics over time. Based on these estimates, the average expected per-beneficiary spending growth was calculated for 2008-2011, 2012-2015, and 2016-2018. The difference between expected and actual spending growth was calculated to determine what share of the spending growth change is attributable to each factor.

As a sample calculation, the difference in per-beneficiary spending growth between the two periods of 2008-2011 and 2012-2015 was a 1.5 percentage point decline. This difference was calculated by summing the differences in growth in payment rates, payment reductions under sequestration, growth in demand by beneficiaries (looking at changes in demand by age, % female, chronic conditions, and % with only Part A) between the two periods 2008-2011 and 2012-2015 (Table 1).

The average spending growth was sequentially calculated using VRDC data on spending per fee-for-service beneficiary every year between 2007-2018. For the 2008-2011 and 2012-2015 periods, the annual spending growth per beneficiary was 3.3 and –0.1, respectively. The difference between the two periods was (–0.1) – 3.3 = –3.4. Two examined contributors to the spending slowdown that were growth in payment rates and payment reductions under sequestration. Growth in payment rates were calculated using the VRDC data on annual CPI-adjusted spending. For period 2008-2011, the CPI growth rates of 2008-2010 were averaged to result in an annual average of 2.4. For period 2012-2015, the CPI growth rates of 2012-2015 were averaged to result in an annual average of 1.6. The difference of these two annual averages resulted in 1.6 – 2.4 = –0.8.

© 2022 Buntin MB et al. *JAMA Health Forum.*

Payment reductions under sequestration were calculated by finding the difference between the average CPI annual average sequential growth and CPI annual average growth calculated from the annual CPI-adjusted spending and CPI sequential growth data provided by the VRDC for each specified time period. For the 2008-2011 and 2012-2015 periods, the differences were 0.0 and –0.5, respectively. The difference in these differences is (–0.5) – 0 = –0.5.

Finally, growth in demand by beneficiaries was also examined as a contributor to the annual growth in Medicare Parts A and B per-beneficiary spending. Changes in age, % female, chronic conditions, and with only Part A were examined. The VRDC annual data on growth in these categories were used to calculate the changes in demand by beneficiaries. The differences between the 2008-2011 and 2012-2015 periods were calculated. All of these differences were summed to calculate the percentage point difference in spending growth explained for the period: (–0.8)+(–0.5) +(–0.3) + 0 + 0.1 + 0 = –1.5.

By comparing the percentage points associated with annual spending growth per beneficiary and the percentage points associated with different factors contributing to annual growth, changes in payment rates and beneficiary characteristics explained 44% (-1.5 / -3.4 = 0.44) of the observed slowdown.

© 2022 Buntin MB et al. *JAMA Health Forum.*

References

1. CMS Chronic Condition Warehouse (CCW): CCW Condition Algorithms. Published online February 2019. https://www.ccwdata.org/documents/10280/19139421/ccw-chronic-condition-algorithms.pdf

2. Hartman M, Martin AB, Espinosa N, Catlin A, The National Health Expenditure Acc. National Health Care Spending In 2016: Spending And Enrollment Growth Slow After Initial Coverage Expansions. *Health Affairs*. 2018;37(1):150-160. doi:10.1377/hlthaff.2017.1299

3. Davis P. *Medicare and Budget Sequestration*.; 2021. Accessed May 13, 2021. https://fas.org/sgp/crs/misc/R45106.pdf

4. Conti R, Crosson F, Coukell A, Frank R. Reform Medicare Part B To Improve Affordability And Equity. *Health Affairs Blog*. Published online June 25, 2021. doi:10.1377/hblog20210622.349716

5. Nguyen N, Sheingold S. *Medicare Part B Drugs: Trends in Spending and Utilization, 2006-2017*. Office of the Assistant Secretary for Planning and Evaluation, U.S. Department of Health and Human Services; 2020. https://aspe.hhs.gov/sites/default/files/private/pdf/264416/Part-B-Drugs-Trends-Issue-Brief.pdf

© 2022 Buntin MB et al. *JAMA Health Forum.*

eTable 2. Beneficiary Demographics Characteristics (Used in Model of Demand for Services), 2007-2018

	2007	2008	2009	2010	2011	2012	2013	2014	2015	2016	2017	2018
n=	30,826,752	30,595,908	30,577,709	30,926,057	31,621,731	32,236,438	32,640,429	32,862,469	33,286,961	34,024,645	34,366,382	34,608,390
**Age (%)**												
**65-69**	29.0	29.7	30.2	30.5	31.5	32.5	33.0	33.9	34.7	34.6	34.2	34.0
**70-74**	22.0	22.1	22.1	22.1	22.1	22.3	22.6	22.7	22.7	23.4	24.3	24.8
**75-79**	18.6	18.0	17.6	17.3	16.9	16.6	16.6	16.3	16.2	16.2	16.4	16.7
**80-84**	15.1	14.8	14.4	14.2	13.7	13.1	12.6	12.2	11.8	11.6	11.4	11.3
**85+**	15.3	15.4	15.7	15.8	15.7	15.4	15.2	14.9	14.6	14.2	13.7	13.3
**Sex (%)**												
**M**	43.0	43.4	43.6	43.8	44.1	44.4	44.7	45.1	45.3	45.5	45.7	45.9
**F**	57.0	56.6	56.4	56.2	55.9	55.6	55.3	54.9	54.7	54.5	54.3	54.1
**CC (%)**												
**0**	10.5	11.1	11.6	11.8	12.9	13.7	14.0	14.5	14.8	15.1	15.6	16.0
**1-3**	3.2	3.2	3.1	3.0	3.2	3.3	3.3	3.3	3.3	3.2	3.2	3.3
**4+**	86.4	85.7	85.3	85.1	83.9	83.0	82.7	82.2	81.9	81.7	81.2	80.7

Source: Authors' calculations using 100% Master Beneficiary Summary File information for all fee-for-service beneficiaries aged 65 and over.

Note: CC(%) indicates the percentage of beneficiaries that have chronic conditions, grouped into 3 categories: 0, 1-3, 4 or more.

© 2022 Buntin MB et al. *JAMA Health Forum.*
